# Heparin sulfate is the attachment factor of duck Tembus virus on both BHK21 and DEF cells

**DOI:** 10.1186/s12985-019-1246-1

**Published:** 2019-11-12

**Authors:** Shaoxiong Wu, Zhen Wu, Yuanyuan Wu, Tao Wang, Mingshu Wang, Renyong Jia, Dekang Zhu, Mafeng Liu, Xinxin Zhao, Qiao Yang, Ying Wu, Shaqiu Zhang, Yunya Liu, Ling Zhang, Yanling Yu, Leichang Pan, Shun Chen, Anchun Cheng

**Affiliations:** 1 Research Center of Avian Disease, College of Veterinary Medicine, Sichuan Agricultural University, Wenjiang District, Chengdu City, 611130 Sichuan Province China; 2 Institute of Preventive Veterinary Medicine, College of Veterinary Medicine, Sichuan Agricultural University, No. 211 Huimin Road, Wenjiang District, Chengdu City, 611130 Sichuan Province China; 3 Key Laboratory of Animal Disease and Human Health of Sichuan Province, Wenjiang District, Chengdu City, 611130 Sichuan Province China

**Keywords:** Glycosaminoglycans, Heparin sulfate, Chondroitin sulfate, Attachment factor, Entry

## Abstract

**Background:**

Duck tembusu virus (DTMUV, genus Flaviviruses, family Flaviviridae) is an emerging flavivirus that can infect a wide range of cells and cell lines in vitro, though the initial step of virus invasion remains obscure.

**Methods:**

In this study, drug treatments that including heparin, chondroitin sulfate, heparinase I, chondroitinase ABC and trypsin were applied to detect the influence of DTMUV absorption, subsequently, the copy number of viral genome RNA was analyzed by quantitative real-time PCR. The inhibition process of viral absorption or entry by heparin was determined by western blotting, and the cytotoxicity of drug treated cells was detected by cell counting kit-8.

**Results:**

We found that the desulfation of glycosaminoglycans (GAGs) with sodium chlorate had a significant effect on the adsorption of DTMUV in both BHK21 and DEF cells. Based on this result, we incubated cells with a mixture of DTMUV and GAGs competition inhibitors or pre-treated cells with inhibitors, after incubation with the virus, the NS5 expression of DTMUV and viral titers were detected. The data suggested that heparin can significantly inhibit the absorption of DTMUV in a dose dependent manner but not at the step of viral entry in BHK21 and DEF cells. Meanwhile, heparinase I can significantly inhibit DTMUV attachment step.

**Conclusions:**

Our results clearly proved that heparin sulfate plays an important role in the first step of DTMUV entry, viral attachment, in both BHK21 and DEF cells, which sheds light on the entry mechanism of DTMUV.

## Background

DTMUV is an arthropod-borne flavivirus responsible for the severe decline in egg production in ducks. Since 2010, a newly emerging disease characterized by egg-drop syndrome has occurred in ducks in China. Egg production in sick ducks was seriously decreased within 2 weeks after disease onset. The infected ducks mainly showed clinical symptoms such as depression, loss of appetite, growth retardation, even paralysis or death. The disease has caused serious economic losses to the duck industry. Additionally, DTMUV can exhibit pathogenicity to Kunming mice by intracerebral inoculation [1–5]. DTMUV is similar to other flaviviruses, consisting of an 11-kb positive-sense single-stranded RNA genome composed of one long open-reading frame, encoding three structural proteins (capsid [C]; pre-membrane [prM], which is post-translationally cleaved to produce the pr and M proteins; and envelope [E]) and seven non-structural proteins (NS1, NS2A, NS2B, NS3, NS4A, 2KNS4B and NS5) [6–8]. Flavivirus invasion into cells is a complex process involving many cellular receptors and viral components [9]. Binding to the cellular surface is the first step for flavivirus entry, which mainly depends on E protein. Moreover, prM and phosphatidylserine (PtdSer) can also recognize some cellular receptors [10]. The crystal structure revealed that flavivirus E protein structures have the following three distinct domains: the N-terminal central β-barrel-shaped domain I; the elongated finger-like domain II, which mediates the low-pH-driven membrane fusion of the viral membrane with the host endosomal membrane, and the C-terminal immunoglobulin-like domain III, which is required for binding to the cellular receptor [11–14].

Flaviviruses are arboviruses that can be transmitted by mosquitoes, and most mammalian cells are susceptible to flavivirus infection. Flavivirus require two different sets of receptors to enter the cell as previously reported [15]. The first step in the flavivirus infectious cell entry pathway is concentration of the virus on the cell surface by attachment factors. Then, the virion bind to the greater affinitive entry receptor and begin to enter the cells [9]. Heparin sulfate proteoglycans (HSPGs) are involved in the adsorption of flaviviruses and comprise a core protein structure and GAGs chains. HSPGs are divided into the following three families according to their core protein structure: glypicans, syndecans and perlecans [16]. Meanwhile, sulfated GAGs, which include heparin sulfate, chondroitin sulfate and dextran sulfate, are expressed on a number of cell types and are utilized as attachment factor by some flaviviruses [17–19]. Flavivirus binding to GAGs is due to the electrostatic interaction of positively charged residues on the surface of the E glycoprotein with negatively charged sulfate groups. Significantly, heparin sulfate, but not other GAGs components, is confirmed as attachment factors for some flaviviruses, such as Dengue Virus (DENV) [20], Yellow fever virus (YFV) [21] and Japanese encephalitis virus (JEV) [22].

DTMUV is a relatively recent virus and has attracted much attention. DTMUV is spread mainly through birds such as ducks and geese, but recent studies have also found that DTMUV can also infect mice [23]. To further understand the molecular mechanisms of DTMUV invasion, DEF and BHK21 cells were used for subsequent experiments. We first proved that GAGs were involved in the adsorption of DTMUV by sodium chlorate desulfated GAGs. We then indicated that heparin but not chondroitin sulfate A inhibited the absorption of DTMUV. Furthermore, we verified the results with corresponding GAG-lyases including heparinase I and chondroitinase ABC. Finally, we found that heparin only affected virus adsorption but did not affect its entry. Additionally, we briefly discussed which DTMUV structures are involved in adsorption and we found that E protein was important for the adsorption by treating DTMUV with trypsin.

## Methods

### Virus, cells and chemical

The DTMUV CQW1 strain was propagated in DEF cells as previously described [2] and the virus titer was 10^5^ TCID_50_/0.1 ml. Baby Hamster Kidney (BHK21) cells were provided by our laboratory and maintained in Dulbecco’s modified Eagle’s medium (DMEM) (Sigma, St. Louis, MO, USA) supplemented with 10% fetal bovine serum (FBS) (Gibco, Gaithersburg, MD, USA), penicillin 100 IU/ml and streptomycin 100 μg/ml at 37 °C in a 5% CO_2_ incubator. Primary duck embryo fibroblast (DEF) cells were obtained from duck embryo and cultured at 37 °C and 5% CO_2_ in DMEM with 10% FBS. Heparin and chondroitin sulfate A were purchase from Solarbio (Beijing, China). Heparinase I, chondroitinase ABC and sodium chlorate were purchased from Sigma (St. Louis, MO, USA). The anti-flavivirus group antigen antibody (MAB10216) was purchased from Merck (New Jersey, USA).

### DTMUV binding assay

BHK21 or DEF cells were grown into monolayers in 12-well cell culture plates and then incubated with 1000TCID_50_ DTMUV in the absence or presence of heparin and chondroitin sulfate A at 4 °C for 1 h. The unbound DTMUV was removed by washing cells three times with PBS. Total DTMUV RNA levels were determined by qRT-PCR.

### Sodium chlorate treatment

The addition of sodium chlorate can block cellular ATP-sulfurylase and sulfate adenyl transferase activities and reduce the sulfation levels of GAGs as previous described [20, 21]. BHK21 cells were cultured for 1 week in low-sulfated culture medium RPMI with 10% FBS containing the different concentration of sodium chlorate or 4 mM sodium sulfate as a supplement. For virus binding, the cells were grown into monolayers in 12-well plates that were incubated with 1000TCID_50_ DTMUV at 4 °C for 1 h. The unbound DTMUV was removed by washing the cells three times with PBS. Total cellular RNA was extracted from infected cells using Trizol Reagent, and DTMUV viral RNA levels were determined by quantitative real-time PCR (qRT-PCR) with mouse β-actin as the endogenous control.

### GAG-lyases treatment

Heparinase I can cleave glycosidic linkages in heparin sulfate on the cellular surface and chondroitinase ABC specifically cleaves glycosidic linkages in chondroitin sulfate A, B and C [24]. BHK21 or DEF cells were grown into monolayers in 12-well cell culture plates and then incubated for 1 h at 37 °C with 300 μl buffer (20 mM Tris-HCl, pH 7.5, 50 mM NaCl, 4 mM CaCl2, and 0.01% BSA) containing heparinase I or 300 μl buffer (50 mM Trizma HCl, pH 8.0, with 60 mM sodium acetate and 0.02% BSA) containing chondroitinase ABC. The cells were washed with PBS three times and then incubated with 1000TCID_50_ DTMUV at 4 °C for 1 h. The unbound DTMUV was removed by washing the cells three times with PBS. Total DTMUV RNA levels were determined by qRT-PCR.

### DTMUV RNA levels were determined by qRT-PCR

The cellular total RNA was extracted with the Trizol Reagent according to the manufacturer’s instructions. Then 1 μg of RNA per sample was synthesized into cDNA using a One Step TB Green™ PrimeScript™ RT-PCR Kit II (Takara, Dalian, China). Subsequently, relative mRNA levels were quantified using a SYBR Green qPCR kit (Abm, Richmond, BC, Canada) and a real-time cycler (CFX96 Bio-Rad, Hercules, CA, USA). The primers used for qRT-PCR were as described previously [25]. The reaction conditions were as follow: 94 °C for 3 min, followed by 40 cycles of 94 °C for 10 s and 63 °C for 1 min.

### Fifty percent tissue culture infectious dose (TCID_50_) assay

Viral titers were determined using an endpoint dilution assay. Briefly, BHK21 cells in 96-well tissue culture plates were infected with serial 10-fold dilutions of DTMUV in eight replicates. The plates were incubated for 144 h at 37 °C. The viral titers were calculated using the Reed–Muench method.

### Western blotting

Total cellular proteins were boiled in 6x protein loading buffer before separation by 10% SDS-PAGE electrophoresis. Then, the proteins were transferred onto polyvinylidene fluoride (PVDF) membranes (Millipore, Bedford, MA, USA). The membranes were blocked with 5% skim milk in TBST at 37 °C for 1 h. Subsequently, The membranes were incubated with mouse anti-NS5 polyclonal antibodies provided our lab or anti-β-actin antibody (Ruiying Biological, Suzhou, China) overnight at 4 °C. Then, horseradish peroxidase (HRP)-conjugated goat anti-mouse IgG antibody was used as a secondary antibody. Finally, the proteins were visualized by chemiluminescence using an ECL kit (Bio-Rad).

### Cell cytotoxicity assay by cell counting kit-8

DEF and BHK21 cells were seeded into 96-well microtiter plates. After 1 day, 100 μl of spent medium was replaced with an equal volume of fresh medium containing 10% CCK8 (MedChemExpress, New Jersey, USA). Then, the cells were incubated at 37 °C for 2 h. The optical density (OD) at 450 nm was measured with a microplate spectrophotometer and was proportional to the number of viable cells in the wells.

### Statistical analysis

The unpaired Student’s *t*-test (GraphPad Prism software) was used to determine the statistical significance of the differences between the experimental groups. Error bars represented the standard error of the mean. The *p* value < 0.05 was considered statistically significant, and the degree of significance was indicated as follows: * *p* < 0.05, ** *p* < 0.01, and *** *p* < 0.001.

## Result

### Desulfation treatment on DTMUV attachment

**Fig. 1:**
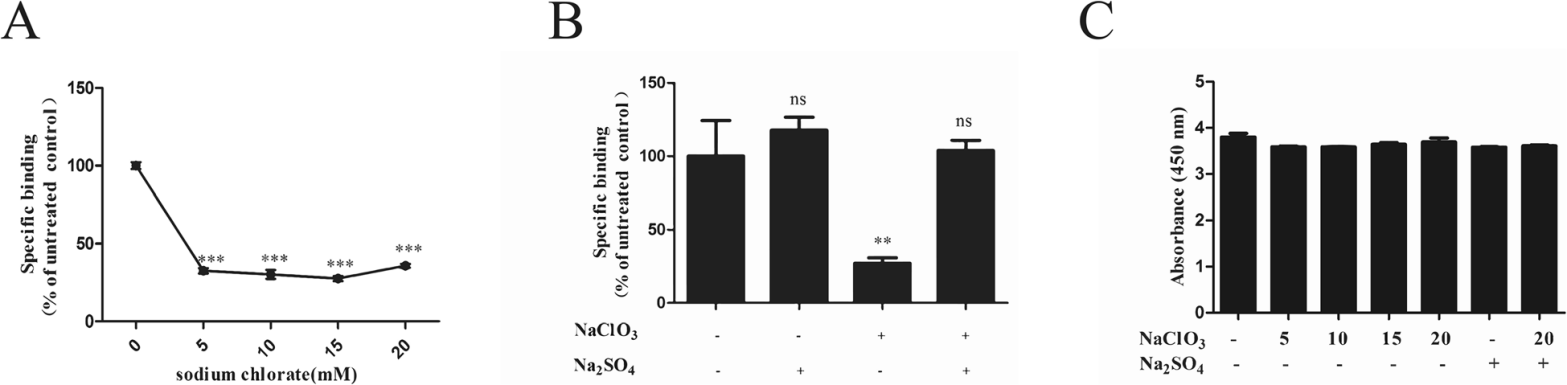
Effects of desulfation on DTMUV attachment. BHK21 cells were treated with different concentrations of sodium chlorate (**a**) for 1 week and then the cells were used for DTMUV binding assays. BHK21 cells were treated with 20 mM sodium chlorate for 1 week in the presence or absence of 20 mM sodium chlorate or 4 mM sodium sulfate (**b**) in RPMI medium containing 10% FBS and then the cells were used for DTMUV binding assays. **c** After treating the cells with sodium chlorate or sodium sulfate, the cell cytotoxicity was tested using a CCK8 kit. The data shown here the average and standard error of four independent experiments. The results of each treatment were compared to their untreated control by Student’s *t* test, *P<0.05, **P<0.01, and ***P<0.001, ns means not significant

To examine whether GAGs affect DTMUV absorption, BHK21 cells were treated with different concentrations of sodium chlorate or 4 mM sodium sulfate as a supplement. We found that the virus adsorption capacity significantly decreased, even at low concentrations such as 5 mM sodium chlorate (Fig. 1a). When BHK21 cells were treated with 4 mM sodium sulfate as a supplement, the virus adsorption capacity did not significantly decrease (Fig. 1b). GAGs desulfation had a significant effect on DTMUV adsorption, indicating that they were involved in the DTMUV adsorption process. Finally, the cells treated with sodium chlorate or sodium sulfate showed no significant cytotoxicity (Fig. 1c).

### Effects of heparin and chondroitin sulfate on DTMUV attachment

**Fig. 2:**
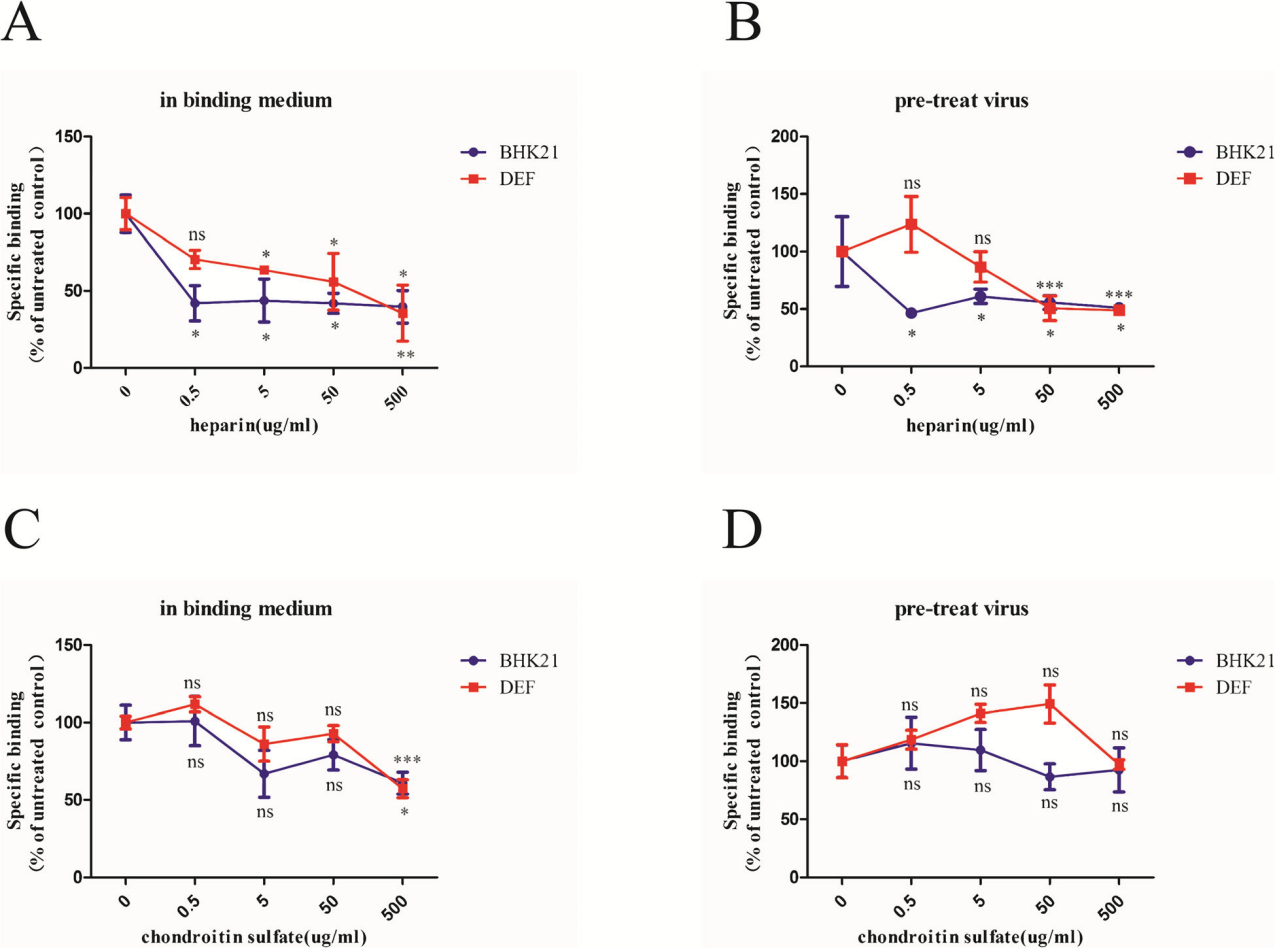
Effects of heparin and chondroitin sulfate on DTMUV attachment. Monolayer BHK21 or DEF cells were treated with a mixture of 1000TCID_50_ DTMUV and different concentrations of heparin (**a**) or chondroitin sulfate A (**c**) at 4 °C for 1 h. Then the unbound virus was removed by washing three times with PBS. DTMUV was pre-treated with different concentration of heparin (**b**) or chondroitin sulfate A (**d**) at 37 °C for 1 h and then incubated with BHK21 or DEF cells at 4 °C for 1 h. After binding, the unbound virus was removed by washing three times with PBS. The data show here the average and standard error of four independent experiments. The results of each treatment were compared to their untreated control by Student’s *t* test, *P<0.05, **P<0.01, and***P<0.001, ns means not significant

To determine the DTMUV attachment factors, we tested two well-known GAGs with cell-surface related receptors for virus binding. The results suggested that incubating cells with a mixture of virus and heparin (Fig. 2a) or pre-treating cells with heparin at 4 °C for 1 h (Fig. 2b), reduced the amount of attached DTMUV in a dose-dependent manner. The decrease in the adsorbed DTMUV reached a maximum in the 500 μg/ml heparin-treated group. Compared with DEF cells, the effect of heparin on virus adsorption was more obvious on BHK21 cells. However, we found that DTMUV absorption decreased significantly when both DEF and BHK21 cells were incubated with a mixture of virus and 500 μg/ml chondroitin sulfate A (Fig. 2c). Additionally, chondroitin sulfate did not inhibited the absorption of DTMUV even after pre-treating the cells at the 500 μg/ml concentration (Fig. 2d). The results indicated that heparin, but not chondroitin sulfate A, can significantly inhibit the absorption of DTMUV. These results indirectly proved that heparin sulfate participate in the DTMUV adsorption process.

### Effects of GAG-lyases on DTMUV attachment

**Fig. 3:**
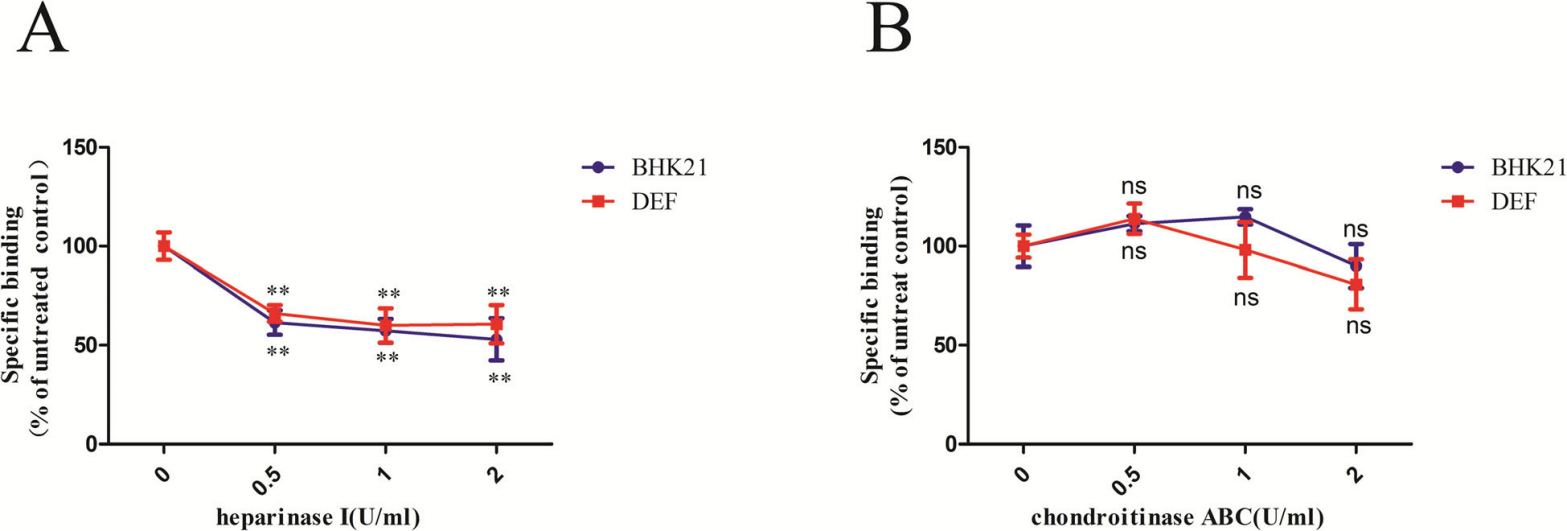
Effects of the GAG-lyases treatment. Monolayer BHK21 and DEF cells were treated with different concentration of heparinase I (**a**) or chondroitinase ABC (**b**) in buffer and then incubated with 1000TCID_50_ DTMUV at 4 °C for 1 h. The unbound virus was removed by washing three times with PBS. The data show here the average and standard error of four independent experiments. The results of each treatment were compared to their untreated control by Student’s *t* test, *P<0.05, **P<0.01, and***P<0.001, ns means not significant

To further confirm that heparin sulfate plays a crucial role in DTMUV attachment, monolayer BHK21 and DEF cells were pre-treated with different concentrations of heparinase I (specific for heparin sulfate) and chondroitinase ABC (specific for chondroitin sulfate ABC) at 37 °C for 1 h, and then incubated with DTMUV. The results (Fig. 3a) showed that heparinase I can significantly inhibit DTMUV binding to BHK21 cells at a concentration of 0.5 U/ml. Interestingly, the downward trend is more obvious in BHK21 cells than DEF cells. Heparinase I showed a 40% inhibitory effect on viral attachment to DEF at 2 U/ml, whereas the amount of DTMUV attachment decreased by 49% to BHK21 cells compared with the control group. In contrast, chondroitinase ABC had no significant effect on DTMUV attachment to either BHK21 or DEF cells (Fig. 3b). The results indicated that heparinase I, but not chondroitinase ABC, can significantly inhibit the absorption of DTMUV both on BHK21 and DEF cells. These results proved that heparin sulfate was involved in DTMUV attachment.

### Heparin inhibits DTMUV attachment but not entry

**Fig. 4:**
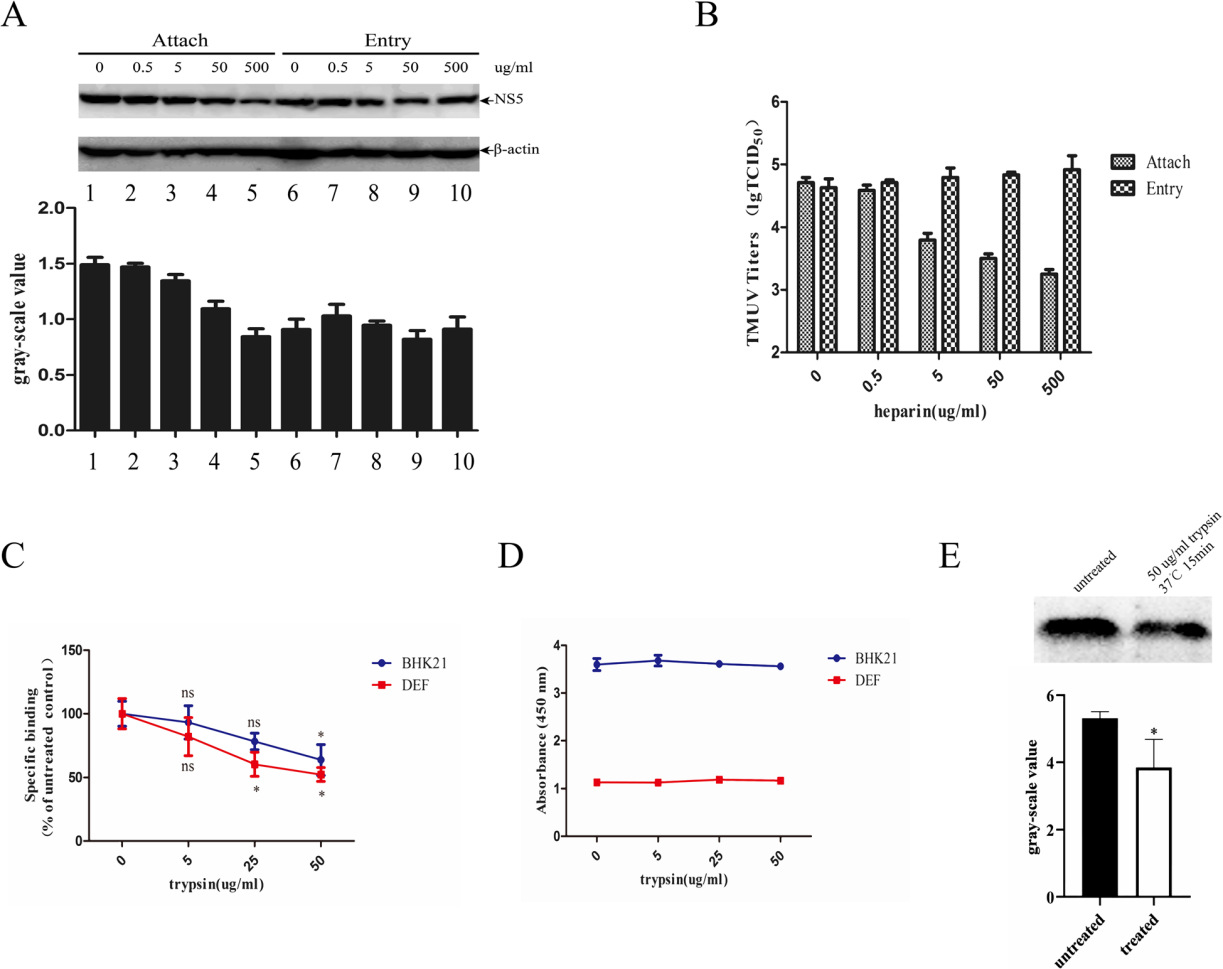
Effects of Heparin on DTMUV attachment and entry. The effect of heparin on DTMUV binding was determined by incubating DEF cells with 1000TCID_50_ DTMUV at 4 °C for 1 h with different concentrations of heparin. The unbound DTMUV was removed by washing the cells three times with PBS, after which the cells were incubated with cell culture medium at 37 °C for 24 h. To determine the effect of heparin on DTMUV entry, DEF cells were incubated with DTMUV at 4 °C for 1 h and then the unbound DTMUV was removed by washing the cells three times with PBS. Subsequently, the DEF cells were incubated with culture medium containing the different concentrations of heparin at 37 °C for 5 h. Culture medium was replaced with fresh medium without heparin. At 24 h post-infection at 37 °C, the NS5 protein levels in the DTMUV-infected DEF cells were detected by Western blotting (**a**) and quantification was reported as gray-scale value. **b** DTMUV titers in the supernatants were detected by TCID50. **c** DTMUV was treated with different concentration of trypsin at 37 °C for 15 min and then incubated with monolayer BHK21 or DEF cells at 4 °C for 1 h. **d** After treating the cells with different concentrations of trypsin at 37 °C for 15 min, the cell cytotoxicity was tested using a CCK8 kit. **e** 1000TCID_50_ DTMUV was concentrated by ultracentrifugation. and then the virus treated with 50 μg/ml trypsin at 37 °C for 15 min. The virus was subjected to SDS-PAGE, the E protein was detected by anti-flavivirus group antigen antibody and quantification was reported as gray-scale value. Values shown represent means from 4 independent experiments

To confirm whether heparin sulfate affects the adsorption or entry of DTMUV, DEF cells were incubated with DTMUV at 4 °C for 1 h in the presence of various concentrations of heparin. Unbound virus was removed by washing the cells with PBS, and then the cells were incubated in medium at 37 °C. At 24 h post-infection, the DTMUV in lysed cell was detected by Western-blotting using an anti-NS5 antibody (Fig. 4a) and DTMUV in the supernatants was titrated by TCID_50_ (Fig. 4b). The results showed that both DTMUV NS5 expression and the viral titers were decreased.

To determine the effects of heparin on DTMUV entry, DEF cells were incubated with DTMUV at 4 °C for 1 h and the unbound virus was removed by washing the cells with PBS. The cells were then incubated in culture medium with different concentrations of heparin at 37 °C for an additional 5 h. Then, the medium was replaced with fresh culture medium and the cells were incubated at 37 °C. As expected, there was no significant decrease in the NS5 protein levels or DTMUV titer compared with the control group. Collectively, these results showed that heparin only affects DTMUV adsorption and does not affect to its cellular entry.

### Trypsin treatment on DTMUV attachment

Previous investigations had suggested that the DENV envelope protein was sensitive to proteases, indicating that trypsin treatment can suppress virus attachment [20]. To determine the effects of trypsin on DTMUV attachment, DTMUV was pre-treated with different concentrations of trypsin and then added to monolayer BHK21 cells. The results (Fig. 4c) showed that trypsin had no effect on DTMUV adsorption at 5 μg/ml. However, both BHK21 and DEF cells showed significant inhibition of attachment when using trypsin at 50 μg/ml concentrations. Additionally, the cells treated with trypsin had no significantly cytotoxicity (Fig. 4d). Then we directly treated the virion with trypsin, these results showed the DTMUV envelope protein can be degraded by trypsin. These indicated that the envelope protein mainly is involved in virus adsorption.

## Discussion

Invading the host cell is the first step in the flavivirus infection cycle, and they can utilize multiple receptors to initiate infection [17]. Virion is first concentrated on the cell surface by binding to attachment factors, and then they are introduced into the endocytosis pathway by a large number of different types of entry receptors, which are internalized into the cell by clathrin-mediated endocytosis [10, 26]. Finally, the low-pH environment within endosomes interacts within the E glycoprotein leading to membrane fusion of the viral membrane with the endosomal membrane, subsequently promoting nucleocapsid release into the cell cytosol [17]. Flaviviruses can infect different animals and spread widely. The identification of the flavivirus receptor will allow us to further explore the mechanism of its cross-host transmission. DTMUV, as a member of flaviviruses, can be transmitted by duck and geese and infect mice by intracranial injection [4, 23]. However, the mechanism of cross-host transmission has remained obscure.

Several studies have indicated that flaviviruses build initial contacts with the host cell by binding to GAGs, such as heparin sulfate and chondroitin sulfate [10]. GAGs are long, unbranched, sulfated polysaccharides that are exposed on the cell surfaces of all tissues and are primarily involved in electrostatic interactions with concentrated virion at the cell surface [27, 28]. To determine the DTMUV attachment factors on BHK21 and DEF cells, we compared the effects of GAGs desulfation (Fig. 1), competitive inhibitors (Fig. 2), and GAG-lyses (Fig. 3) on DTMUV adsorption. These results clearly indicated that heparin sulfate but not chondroitin sulfate was involved in DTMUV adsorption on both BHK21 and DEF cells. Heparin sulfate may be required to concentrate virion at the cell surface. Additionally, heparin sulfate not only initiates the infection by some flaviviruses [16, 19–21] but can also interact with other viruses, such as Sindbis virus [29], Foot-and-mouth disease virus (FMDV) [30], Herpes simplex virus (HSV) [31], Vaccinia virus [32], Venezuelan equine encephalitis virus (VEEV) [33], Papillomavirus [34] and Echovirus [35]. Additionally, Watterson found that interaction between DENV and heparin sulfate occurred through two lysine residues located in domain III of E protein by site-directed mutagenesis studies [36]. Meanwhile, studies have indicated that N- and 6-O-sulfation but not 2-O-sulfation are important for HCV infection by silencing of the enzymes involved in the heparin sulfate biosynthesis pathway [9].

Entry receptors can bind specifically to flaviviruses and direct them to the endocytic pathway after flaviviruses attach to the cell surface [10]. Recently, it has been shown that dendritic cell-specific ICAM-3 grabbing non-integrin (DC-SIGN) and liver/lymph node-specific ICAM-3 grabbing non-Integrin (L-SIGN) that belong to C-type lectins can bind to several flaviviruses, such as Dengue virus [37–39], West Nile virus [40] and Japanese encephalitis virus [41]. Additionally, some investigations have also suggested that the T-cell immunoglobulin and mucin domain (TIM) and TYRO3, AXL and MER (TAM) can enhance flavivirus infection by binding to PtdSer on the virion surface rather than E glycoprotein [42, 43]. To verify the role of heparin sulfate in DTUMV invasion, we compared the effects of heparin on DTMUV adsorption and entry. The results clearly indicated that heparin only affects DTMUV adsorption and does not affect the process of virus internalization. Meanwhile, Philip Hilgard found that DENV E protein is extremely sensitive to trypsin [20]; so we treated DTMUV with trypsin and conducted DTMUV adsorption experiments. We found that trypsin could degrade E protein showing that E protein was mainly involved in virus adsorption. Despite intensive research, little is known about the identity of the cell receptors that mediate flavivirus entry and infection. Many molecules have been described as candidate receptors for flavivirus on different cell types, but their exact role in viral entry remains unclear. So far, there have been several investigations focused on determining the DTMUV entry receptor by virus overlay protein binding assay (VOPBA). Glucose-regulated protein 78 (GRP78), a receptor on BHK21 cells, and heat shock protein A9, a receptor on DF-1 cell, bind to DTMUV to allow cellular entry [44, 45]. However, this is far from enough to thoroughly understand DTMUV invasion mechanism in cells and even in animals, which requires further research.

## Conclusion

Heparin sulfate, a component of GAGs exposed on the cell surface, is involved in DTMUV attachment but not entry. Moreover, heparinase I but not chondroitinase ABC can inhibit DTMUV attachment. Thus, our results may provide a molecular explanation for the pathogenesis of DTMUV.

## Data Availability

The datasets analyzed in this study are available from the corresponding author upon reasonable request.

## Abbreviations

DC-SIGN: Dendritic Cell -specific ICAM-3 grabbing non-integrin
DENV: Dengue Virus
DTMUV: Duck tembusu virus
GAGs: Glycosaminoglycans
GRP78: Glucose-regulated protein 78
HRP: Fluoridehorseradish peroxidase
HSPGs: Heparin sulfate proteoglycans
JEV: Japanese encephalitis virus
L-SIGN: Liver/Lymph Node-Specific ICAM-3 Grabbing Non-Integrin
PtdSer: Phosphatidylserine
PVDF: Polyvinylidene
qRT-PCR: Real-time PCR
TAM: TYRO3, AXL and MER
TCID_50_: Fifty percent tissue culture infectious dose
TIM: T-cell immunoglobulin and mucin domain
VOPBA: Virus overlay protein binding assay
YFV: Yellow fever virus

## References

[1] Yan P, Zhao Y, Zhang X, Xu D, Dai X, Teng Q, Yan L, Zhou J, Ji X, Zhang S, Liu G, Zhou Y, Kawaoka Y, Tong G, Li Z (2011). An infectious disease of ducks caused by a newly emerged Tembusu virus strain in mainland China. Virology.

[2] Zhu K, Huang J, Jia R, Zhang B, Wang M, Zhu D, Chen S, Liu M, Yin Z, Cheng A (2015). Identification and molecular characterization of a novel duck Tembusu virus isolate from Southwest China. Arch Virol.

[3] Yan Z, Shen H, Wang Z, Lin W, Xie Q, Bi Y, Chen F (2017). Isolation and characterization of a novel Tembusu virus circulating in Muscovy ducks in South China. Transbound Emerg Dis.

[4] Zhang W, Chen S, Mahalingam S, Wang M, Cheng A (2017). An updated review of avian-origin Tembusu virus: a newly emerging avian Flavivirus. J Gen Virology.

[5] Ti J, Zhang M, Li Z, Li X, Diao Y (2016). Duck Tembusu virus exhibits pathogenicity to Kunming mice by Intracerebral inoculation. Front Microbiol.

[6] Liu M, Liu C, Li G, Li X, Yin X, Chen Y, Zhang Y (2012). Complete genomic sequence of duck flavivirus from China. J Virol.

[7] Yu G, Lin Y, Tang Y, Diao Y. Evolution of Tembusu virus in ducks, chickens, geese, sparrows, and mosquitoes in northern China. Viruses. 2018;10:485–94. 10.3390/v10090485 PMC6164154 30201873

[8] Chen P, Liu J, Jiang Y, Zhao Y, Li Q, Wu L, He X, Chen H (2014). The vaccine efficacy of recombinant duck enteritis virus expressing secreted E with or without PrM proteins of duck tembusu virus. Vaccine.

[9] Xu Y, Martinez P, Seron K, Luo G, Allain F, Dubuisson J, Belouzard S (2015). Characterization of hepatitis C virus interaction with heparan sulfate proteoglycans. J Virol.

[10] Perera-Lecoin M, Meertens L, Carnec X, Amara A (2013). Flavivirus entry receptors: an update. Viruses.

[11] Dai L, Song J, Lu X, Deng YQ, Musyoki AM, Cheng H, Zhang Y, Yuan Y, Song H, Haywood J, Xiao H, Yan J, Shi Y, Qin CF, Qi J, Gao GF (2016). Structures of the Zika virus envelope protein and its complex with a Flavivirus broadly protective antibody. Cell Host Microbe.

[12] Pokidysheva E, Zhang Y, Battisti AJ, Bator-Kelly CM, Chipman PR, Xiao C, Gregorio GG, Hendrickson WA, Kuhn RJ, Rossmann MG (2006). Cryo-EM reconstruction of dengue virus in complex with the carbohydrate recognition domain of DC-SIGN. Cell.

[13] Das S, Laxminarayana SV, Chandra N, Ravi V, Desai A (2009). Heat shock protein 70 on Neuro2a cells is a putative receptor for Japanese encephalitis virus. Virology.

[14] Rodenhuis-Zybert IA, Moesker B, da Silva Voorham JM, van der Ende-Metselaar H, Diamond MS, Wilschut J, Smit JM (2011). A fusion-loop antibody enhances the infectious properties of immature flavivirus particles. J Virol.

[15] Rey FA, Stiasny K, Heinz FX (2017). Flavivirus structural heterogeneity: implications for cell entry. Current Opinion Virology.

[16] Lin X (2004). Functions of heparan sulfate proteoglycans in cell signaling during development. Development.

[17] Smit JM, Moesker B, Rodenhuis-Zybert I, Wilschut J (2011). Flavivirus cell entry and membrane fusion. Viruses.

[18] Nakamura M, Uehara Y, Asada M, Suzuki M, Imamura T (2013). Sulfated glycosaminoglycan-assisted receptor specificity of human fibroblast growth factor (FGF) 19 signaling in a mouse system is different from that in a human system. J Biomol Screen.

[19] Su CM, Liao CL, Lee YL, Lin YL (2001). Highly sulfated forms of heparin sulfate are involved in japanese encephalitis virus infection. Virology.

[20] Hilgard P, Stockert R (2000). Heparan sulfate proteoglycans initiate dengue virus infection of hepatocytes. Hepatology.

[21] Germi R, Crance JM, Garin D, Guimet J, Lortat-Jacob H, Ruigrok RW, Zarski JP, Drouet E (2002). Heparan sulfate-mediated binding of infectious dengue virus type 2 and yellow fever virus. Virology.

[22] Liu H, Chiou SS, Chen WJ (2004). Differential binding efficiency between the envelope protein of Japanese encephalitis virus variants and heparan sulfate on the cell surface. J Med Virol.

[23] Li S, Li X, Zhang L, Wang Y, Yu X, Tian K, Su W, Han B, Su J (2013). Duck Tembusu virus exhibits neurovirulence in BALB/c mice. Virol J.

[24] Misinzo G, Delputte PL, Meerts P, Lefebvre DJ, Nauwynck HJ (2006). Porcine circovirus 2 uses heparan sulfate and chondroitin sulfate B glycosaminoglycans as receptors for its attachment to host cells. J Virol.

[25] Chen S, Wang T, Liu P, Yang C, Wang M, Jia R, Zhu D, Liu M, Yang Q, Wu Y (2019). Duck interferon regulatory factor 7 (IRF7) can control duck Tembusu virus (DTMUV) infection by triggering type I interferon production and its signal transduction pathway. Cytokine.

[26] Hackett BA, Cherry S (2018). Flavivirus internalization is regulated by a size-dependent endocytic pathway. Proc Natl Acad Sci U S A.

[27] Zhang L (2010). Glycosaminoglycan (GAG) biosynthesis and GAG-binding proteins. Progress Mol Biol Translational Sci.

[28] Chen Y, Maguire T, Hileman RE, Fromm JR, Esko JD, Linhardt RJ, Marks RM (1997). Dengue virus infectivity depends on envelope protein binding to target cell heparan sulfate. Nat Med.

[29] Byrnes AP, Griffin DE (1998). Binding of Sindbis virus to cell surface heparan sulfate. J Virol.

[30] Sa-Carvalho D, Rieder E, Baxt B, Rodarte R, Tanuri A, Mason PW (1997). Tissue culture adaptation of foot-and-mouth disease virus selects viruses that bind to heparin and are attenuated in cattle. J Virol.

[31] Shukla D, Liu J, Blaiklock P, Shworak NW, Bai X, Esko JD, Cohen GH, Eisenberg RJ, Rosenberg RD, Spear PG (1999). A novel role for 3-O-sulfated heparan sulfate in herpes simplex virus 1 entry. Cell.

[32] Lin CL, Chung CS, Heine HG, Chang W (2000). Vaccinia virus envelope H3L protein binds to cell surface heparan sulfate and is important for intracellular mature virion morphogenesis and virus infection in vitro and in vivo. J Virol.

[33] Bernard KA, Klimstra WB, Johnston RE (2000). Mutations in the E2 glycoprotein of Venezuelan equine encephalitis virus confer heparan sulfate interaction, low morbidity, and rapid clearance from blood of mice. Virology.

[34] Joyce JG, Tung JS, Przysiecki CT, Cook JC, Lehman ED, Sands JA, Jansen KU, Keller PM (1999). The L1 major capsid protein of human papillomavirus type 11 recombinant virus-like particles interacts with heparin and cell-surface glycosaminoglycans on human keratinocytes. J Biol Chem.

[35] Goodfellow IG, Sioofy AB, Powell RM, Evans DJ (2001). Echoviruses bind heparan sulfate at the cell surface. J Virol.

[36] Watterson D, Kobe B, Young PR (2012). Residues in domain III of the dengue virus envelope glycoprotein involved in cell-surface glycosaminoglycan binding. J General Virology.

[37] Alen MMF, Kaptein SJF, Burghgraeve TD, Balzarini J, Neyts J, Schols D (2009). Antiviral activity of carbohydrate-binding agents and the role of DC-SIGN in dengue virus infection. Virology.

[38] Boonrat T, Timothy HB, Angela G-P, Christine T, Jennifer F, Wellington S, Michael AE, Kovit P, Suttipant S, Deborah LB (2003). DC-SIGN (CD209) mediates dengue virus infection of human dendritic cells. J Exp Med.

[39] Chen ST, Lin YL, Huang MT, Wu MF, Cheng SC, Lei HY, Lee CK, Chiou TW, Wong CH, Hsieh SL (2008). CLEC5A is critical for dengue-virus-induced lethal disease. Nature.

[40] Davis CW, Hai-Yen N, Hanna SL, Sánchez MD, Doms RW, Pierson TC (2006). West Nile virus discriminates between DC-SIGN and DC-SIGNR for cellular attachment and infection. J Virol.

[41] Wang P, Hu K, Luo S, Zhang M, Deng X, Li C, Jin W, Hu B, He S, Li M (2016). DC-SIGN as an attachment factor mediates Japanese encephalitis virus infection of human dendritic cells via interaction with a single high-mannose residue of viral E glycoprotein. Virology.

[42] Meertens L, Carnec X, Lecoin MP, Ramdasi R, Guivel-Benhassine F, Lew E, Lemke G, Schwartz O, Amara A (2012). The TIM and TAM families of Phosphatidylserine receptors mediate dengue virus entry. Cell Host Microbe.

[43] Kuadkitkan A, Wikan N, Fongsaran C, Smith DR (2010). Identification and characterization of prohibitin as a receptor protein mediating DENV-2 entry into insect cells. Virology.

[44] Zhao D, Liu Q, Han K, Wang H, Yang J, Bi K, Liu Y, Liu N, Tian Y, Li Y (2018). Identification of glucose-regulated protein 78 (GRP78) as a receptor in BHK-21 cells for duck Tembusu virus infection. Front Microbiol.

[45] Liu Q, Huang X, Zhao D, Han K, Liu Y, Yang J, Bi K, Li Y (2017). Identification of heat shock protein A9 as a Tembusu virus binding protein on DF-1 cells. Virus Res.

